# Temporal and Spatial Variability of Fungal Structures and Host Responses in an Incompatible Rust–Wheat Interaction

**DOI:** 10.3389/fpls.2017.00484

**Published:** 2017-04-12

**Authors:** Chris K. Sørensen, Rodrigo Labouriau, Mogens S. Hovmøller

**Affiliations:** ^1^Department of Agroecology, Aarhus UniversitySlagelse, Denmark; ^2^Department of Mathematics, Aarhus UniversityAarhus, Denmark

**Keywords:** gene-for-gene, yellow rust, two-photon microscopy, plant resistance, host–pathogen interaction

## Abstract

Information about temporal and spatial variability of fungal structures and host responses is scarce in comparison to the vast amount of genetic, biochemical, and physiological studies of host–pathogen interactions. In this study, we used avirulent wild type and virulent mutant isolates of *Puccinia striiformis* to characterize the interactions in wheat carrying yellow rust *Yr*2 resistance. Both conventional and advanced microscopic techniques were used for a detailed study of morphology and growth of fungal colonies and associated host cell responses. The growth of the wild type isolates was highly restricted due to hypersensitive response (HR, plant cell death) indicated by autofluorescence and change in the shape of the affected plant cells. The host response appeared post-haustorial, but large variation in the time and stage of arrest was observed for individual fungal colonies, probably due to a delay between detection and response. Some colonies were stopped right after the formation of the primary infection hyphae whereas others formed highly branched mycelia. HR was first observed in host cells in direct contact with fungal structures, after which the defense responses spread to adjacent host cells, and eventually led to encasement of the fungal colony. Several cells with HR contained haustoria, which were small and underdeveloped, but some cells contained normal sized haustoria without signs of hypersensitivity. The growth of the virulent mutants in the resistant plants was similar to the growth in plants without *Yr*2 resistance, which is a strong indication that the incompatible phenotype was associated with *Yr*2. The interaction between *P. striiformis* and wheat with *Y*r2 resistance was highly variable in time and space, which demonstrate that histological studies are important for a deeper understanding of host–pathogen interactions and plant defense mechanisms in general.

## Introduction

Plants have developed both passive and active defense systems to protect themselves against pathogens. Pathogens which are able to overcome the passive system consisting of preformed physical and chemical barriers, are confronted by an active two-layered defense system (Dodds and Rathjen, 2010). The first layer of active defense is setup for detection of pathogen-associated molecular patterns resulting from the breakdown of either pathogen or plant structures during infection (Wirthmueller et al., 2013). When this system is effective, the plant is characterized as a non-host to the intruding pathogen. Pathogens seek to bypass the active defense system to gain access to nutrition either by killing or parasitizing the plants. Parasitic pathogens with a biotrophic life style, have evolved to establish basic compatibility with their host by jamming the first layer of active defense with small effector proteins (Toruño et al., 2016). In the second layer of active defense, plant intercellular receptors may recognize these effectors and initiate localized cell death, denoted effector triggered immunity (ETI), i.e., hypersensitive response (HR) that prevent the pathogen from taking up nutrients from the affected plant cells. However, plant–pathogen recognition may be lost in the absence of plant receptors that match pathogen effectors (Wirthmueller et al., 2013). There is mounting evidence that biotrophic pathogens may deliver effectors into the plant cell via specialized structures, haustoria that develop between the cell wall and plasma membrane of host cells (Dodds et al., 2009; Stergiopoulos and de Wit, 2009). It is generally assumed that effector recognition results in HR although there may be significant variation of the response in both time and space (Mur et al., 2008; Wang et al., 2013; Niks et al., 2015). When the pathogen is stopped by an ETI response the interaction is considered incompatible.

Histopathological studies based on techniques like bright field and electron and conventional fluorescence microscopy have proven valuable for a general understanding of the infection biology of plant pathogens and of the cytological changes in individual host and pathogen cells during infection. However, the traditional microscopy techniques have some built-in limitations, which constrain visualizations of structures in intact tissues. In contrast, new techniques like confocal and two-photon microscopy can be used for accurate observations of the host–pathogen interaction in tissue-bound cells (Feijó and Moreno, 2004; Moreno et al., 2006; Minker et al., 2016). This allows precise assessment of temporal and spatial variation of the interactions, which is important for an in-depth elucidation of the underlying mechanisms.

In the present study, we used both traditional and new microscopy techniques for histological investigation of the interactions between the fungal pathogen *Puccinia striiformis* and wheat varieties carrying *Yr*2 resistance, which is associated with ETI-based HR. Macroscopically, *Yr*2 resistance results in chlorotic and necrotic leaf areas containing no or miniscule amounts of sporulation when exposed to an avirulent pathogen isolate. *P. striiformis* causes yellow rust on cereals and wild grasses and it is currently one of the most important diseases in wheat production. It occurs in most parts of the world’s wheat growing areas where it may cause significant yield losses (Wellings, 2011). The pathogen infects its grass host via the stomata, and in compatible interactions it grows semi-systemically in the apoplast between the leaf mesophyll cells (Cartwright and Russell, 1981). Numerous haustoria are produced inside host cells during the expansion of the mycelium (Sørensen et al., 2012). In susceptible host genotypes new spore producing pustules erupts through the leaf epidermis 10–14 days after infection. Several major resistance genes (R-genes) have been utilized in wheat on a large-scale in an attempt to control yellow rust, but in many cases the effect was lost after few years of deployment. This outcome is probably due to strong selection for virulent pathogen genotypes. Virulence is fundamentally believed to arise from mutation in an avirulence gene so that the gene product (effector) is no longer detected by the product of the R-gene (McDonald and Linde, 2002).

Here we used two independent pairs of avirulent wild type and virulent mutant isolates. The mutant isolates, which were collected from field trials designed to select for spontaneous virulence mutants, only differed from the respective wild type in being virulent on wheat varieties carrying *Yr*2 resistance (Sørensen et al., 2013). The objective of the study was to carry out a histological characterization of the temporal and spatial variability of the incompatible interaction between the wild type isolates and wheat varieties with *Yr*2 resistance. The mutant isolates were used as controls to substantiate that the observation for the incompatible interactions were due to the effect of *Yr*2 resistance. The results are discussed in the context of breeding for disease resistance and the perspectives for using histology as a tool to increase our understanding of host–pathogen interactions.

## Materials and Methods

### Wheat Varieties and Pathogen Isolates

Fungal colony growth and host response were assessed in the second leaf of wheat seedlings of two susceptible varieties Avocet S and Cartago, and two varieties carrying *Yr*2-resistance, Skater (*Yr*2, *Yr*32; Hovmøller, 2007) and Heines VII (*Yr*2, *Yr*25, +; Calonnec et al., 1997b). Complementary observations of the resistant *Yr*2 response were conducted on the varieties Heines Peko (*Yr*2, *Yr*6, *Yr*25, +; Calonnec et al., 1997b) and Kalyansona (*Yr*2, +; Singh and Johnson, 1988). Ten seeds were sown in 7 cm × 7 cm × 7 cm pots with a standard peat-based mix with slow release nutrients (Pindstrup Mosebrug A/S). Plants were grown in spore-proof growth cabins, where 50–100 μEm^-2^ s^-1^ of artificial light was applied when daylight <10,000 lux (16 h day/8 h night). Temperatures were set to 17°C day and 12°C at night.

Two wild type *Yr*2-avirulent isolates, DK24/95 and GB75/30, and two *Yr*2-virulent mutant isolates, Mut15/05 and Mut21/06, were used (**Table 1**). The mutant isolates were collected in 2005 and 2006 from inoculated field trials set up for the detection of spontaneous virulence mutants that could reproduce on wheat lines carrying *Yr*2 resistance. Rows of the susceptible wheat variety Anja were inoculated with the wild type isolates. The rows of Anja were flanked by rows of the variety Skater (*Yr*2, *Yr*32), which was resistant to the Danish population of *P. striiformis* at that time. Infections emerging on Skater during the trial were taken to the laboratory for virulence and amplified fragment length polymorphism (AFLP) phenotyping according to Justesen et al. (2002). These tests identified the *Yr*2 virulence mutant isolates, which shared AFLP fingerprint with their respective wild type isolates when screened by 20 AFLP primer combinations producing c. 1400 AFLP fragments (for more details, see Sørensen et al., 2013).

**Table 1:** Virulence phenotypes of the wild type (GB75/30 and DK 24/95) and mutant (Mut15/05 and Mut21/06) isolates of *Puccinia striiformis* used in this study.

	Specific *Yr*-resistance^a^																	
	
Isolate	1	2	3	4	5	6	7	8	9	10	15	17	25	27	32	Sd^b^	Sp^b^	Su^b^
GB75/30	–	–	–	–	–	–	–	–	–	–	–	–	25	–	32	Sd	(Sp)	–
Mut15/05	–	(2)	–	–	–	–	–	–	–	–	–	–	25	–	32	Sd	(Sp)	–
DK24/95	–	–	3	4	–	6	–	–	–	–	–	–	25	–	32	Sd	–	Su
Mut21/06	–	2	3	4	–	6	–	–	–	–	–	–	25	–	32	Sd	–	Su


*^a^Avirulence was indicated by “–” and virulence was defined by infection type (IT) above 6 on a 0–9 scale (McNeal et al., 1971). Parentheses indicate intermediate IT scores of 5–6. ^b^Resistance specificities in Strubes Dickkopf (Sd), Spalding Prolific (Sp), and Suwon/Omar (Su), respectively.*

Spores for experimental use were produced on seedlings of Cartago treated with 0.33% maleic hydrazide acid (maleic hydrazide, Antergon^®^ MH 180, Crompton Registrations Ltd., Birmingham, England). Inoculated plants were incubated in darkness at 10°C for 20–24 h and transferred to the greenhouse in spore-proof growth cabins under the conditions described above. Plants were covered with cellophane bags before the onset of sporulation. Seedlings in individual pots were shaken approximately 48 h before harvest of fresh spores to remove old spores. The harvested spores were used for experiments within the same day.

### Experimental Setup and Sampling

Plants were inoculated when the second leaf was fully expanded 16 days after sowing. Ten pots of each variety were inoculated per isolate. The numbers of plants per pot were trimmed to five of equal size prior to inoculation. Spores were mixed with talc (1:19 w/w) and applied to a 2 cm long area of the second leaf approximately 11 cm below the leaf tip using a camel hair brush (size 1). Plants were sprayed with water and pots placed in trays, covered with lids and incubated as above. Plants were transferred to spore-proof cabins, where pots were randomized between and within four individual cabins. One leaf segment was sampled per pot at 3, 5, 7, and 16 days post inoculation (dpi), resulting in a total of 10 segments per isolate–variety interaction per time point. On day 16, infection types (IT) were assessed on the remaining plants in each pot using a 0–9 scale (McNeal et al., 1971). The experiment was replicated twice at different times of the year. Plants of Heines Kolben and Kalyansona for complementary observation of host responses were inoculated as described above and infected leaves were sampled 1, 3, 5, and 7 dpi.

### Staining and Microscopy

Leaf samples were transferred directly to the laboratory, where staining was done according to Moldenhauer et al. (2006). The segments were fixed and cleared in ethanol:chloroform (3:1, v/v) + 0.15% (v/w) trichloroacetic acid for at least 24 h. After being washed twice in 50% ethanol they were left in 0.05 M NaOH for 30 min. Specimens were then rinsed in water before being submerged in 0.1 M Tris–HCl buffer (pH 5.8) for 30 min. Afterward they were stained for 5 min in 0.1% (w/v) Uvitex 2B (Polysciences Inc.). Following staining, specimens were washed four times in deionized water (DI), one time in 25% glycerol and left overnight in DI. They were stored in 50% glycerol until further use. Whole mounts were prepared and microscopy for determination of colony size and morphology were carried out with a Leica DMR equipped with optics for epifluorescence. Colonies and autofluorescence due to resistance were visualized using UV-1D filter, excitation filter 355–425 and barrier filter 455. Between two and five randomly selected colonies were measured per leaf segment; leaf segments with only one infection site were not considered. Colony dimensions were measured with a calibrated eyepiece micrometer and the size was calculated as largest length × largest width × π/4 (Baart et al., 1991). For the compatible interactions, colonies generally started to overlap at 7 dpi, which reduced the number of undisturbed single colonies, and at 16 dpi the assessment of single colonies was no longer possible. The exact numbers of colonies measured per treatment are shown in Supplementary Figures S1–S4.

Fungal colonies and plant responses for the wild type isolates in the resistant varieties (Skater, Heines VII, Heines Peko, and Kalyansona) were further analyzed using an inverted Zeiss LSM 510 Meta confocal laser scanning microscope. Fungal structures and leaf tissue were excited with laser beams at 720 nm using a Mai Tai two-photon laser and scanned with filters settings BP 435–485 nm. Autofluorescence caused by the resistant reactions were visualized by a 514 nm argon-laser and a 543 nm HeNe1 laser with BP 565–635 nm filter settings. Z-stacks for 3-D projections were collected with 1 μm separation of images. 3-D image projection was performed with Zeiss LSM image browser 4.2.0.121. Further image editing of 3-D projections, such as size adjustments and sharpening, was done with Adobe Photoshop CS5 (Adobe Systems Inc. San Jose, CA, USA).

### Statistics

The temporal growth of colonies were modeled as exponential growth curves (one for each combination of variety and isolate), adjusted using a generalized linear model (see McCullagh and Nelder, 1989; Jørgensen et al., 1996) with gamma-distributed responses, logarithmic link function and a linear predictor containing the number of dpi as a continuous explanatory variable and a factor (i.e., a classification explanatory variable) representing the combination of variety isolate and, additionally, a factor representing the experiment. More precisely, the gamma exponential regression model referred above assumes that colony size Y_iedr_ of the *r*th replicate of the *i*th combination of variety and isolate obtained from the *e*th experiment at the *d*th dpi is gamma distributed and has expected value |E(Y_iedr_)| such that

$$\text{log}\{\text{E}\left({\text{Y}}_{\text{iedr}}\right)\}=\mu_{i}+\beta_{i}d+\gamma_{e}$$

or equivalently,

$$\text{E}({\text{Y}}_{\text{iedr}})=\text{exp}\left(\gamma_{\text{e}}\right)\text{exp}\left(\mu_{i}\right)\text{exp}\left(\beta_{i}d\right)$$

The parameters β_i_ represent the rate of exponential growth of the colonies specific to the *i*th combination of variety and isolate. The model was inferred using the function glm of the software R (R Core Team, 2013) Likelihood ratio tests were used to test differences of the growth rate between combinations of variety and isolate. The pairwise comparisons (**Figure 2**) were adjusted for multiple comparisons by the false discovery rate method (Benjamini and Hochberg, 1995; Benjamini and Yekutieli, 2001).

The observed distributions of the colony sizes at different days after inoculation (dai) were compared for each combination of variety and isolate (**Table 2**) using a one-sided Kolmogorov–Smirnov test (Conover, 1971). The null hypotheses of equality of colony size distributions at two different dpi were tested against the alternative hypothesis, that the distribution of the lesion sizes observed later was stochastically larger than the distribution of lesion sizes observed earlier (Lehmann, 1955).

**Table 2:** Minimum, maximum, and variance for colony size of the *Puccinia striiformis* isolates GB75/30 and DK24/95 in seedling leaves of two wheat varieties Skater and Heines VII which carries *Yr*2 resistance, at 3, 5, 7, and 16 days post inoculation (dpi). Functions for colony size frequency distribution were compared at individual time points by (one-sided) Kolmogorov–Smirnov tests.

		*P*-values^a^					
					
Isolate—Cultivar	Time (dpi)	5 dpi	7 dpi	16 dpi	Variance	Minimum (mm^2^)	Maximum (mm^2^)
GB75/30—Skater	3	*P* < 0.0001	*P* < 0.0001	*P* < 0.0001	0.00001	0.0005	0.020
	5	–	*P* < 0.0001	*P* < 0.0001	0.00305	0.0003	0.412
	7	–	–	*P* = 0.0304	0.00323	0.0013	0.312
	16	–	–	–	0.02429	0.0004	1.443
GB75/30—Heines VII							
	3	*P* < 0.0001	*P* < 0.0001	*P* < 0.0001	0.00001	0.0001	0.014
	5	–	*P* = 0.0351	*P* < 0.0001	0.01028	0.0004	0.551
	7	–	–	*P* = 0.0143	0.02629	0.0005	0.758
	16	–	–	–	0.08921	0.0009	3.760
DK24/95—Skater							
	3	*P* < 0.0001	*P* < 0.0001	*P* < 0.0001	0.00002	0.0006	0.021
	5	–	*P* = 0.0087	*P* = 0.0067	0.00178	0.0004	0.248
	7	–	–	*P* = 0.6159	0.00717	0.0004	0.567
	16	–	–	–	0.36388	0.0002	5.133
DK24/95—Heines VII							
	3	*P* < 0.0001	*P* < 0.0001	*P* < 0.0001	0.00003	0.0004	0.023
	5	–	*P* < 0.006	*P* < 0.0001	0.00717	0.0003	0.447
	7	–	–	*P* = 0.0023	0.15101	0.0004	2.189
	16	–	–	–	0.79300	0.0004	6.311


*^a^*P*-values from the Kolmogorov–Smirnov test for testing the null hypothesis of equality of the colony size distributions at different time points [days post-inoculation (dpi)] against the one-sided alternative hypothesis that the distribution of the corresponding lesion sizes observed later is stochastically larger than the distribution of the lesion size observed earlier.*

## Results

### Infection Type

The four isolates were previously tested for virulence phenotype on wheat differential sets, which showed that the mutant isolates only differed from their wild type with respect to virulence on host plants carrying *Yr*2 resistance (**Table 1**).

**FIGURE 1:**
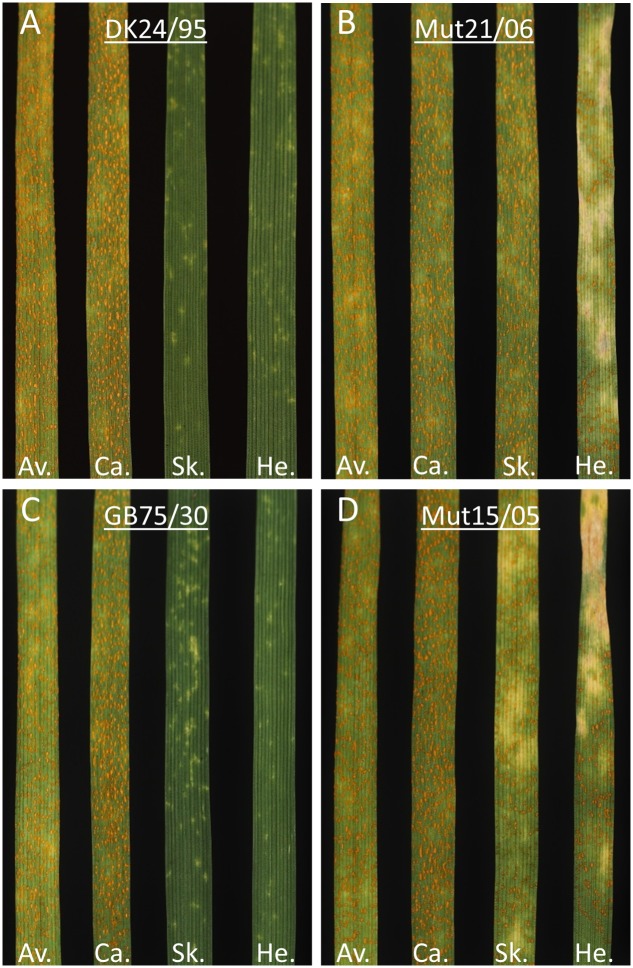
**Infection types (IT) of two pair of *Puccinia striiformis* wild type and mutant isolates DK24/95–Mut21/06 and GB75/30–Mut15/05 on seedlings of two susceptible wheat varieties Avocet S (Av.) and Cartago (Ca.) and on two varieties Skater (Sk.) and Heines VII (He.) with the *Yr*2 resistance gene.** The presence of chlorosis and necrosis on the leaves is denoted as “c” and “n”, respectively. **(A)** Isolates DK24/95 gave the following IT: Av = IT 8, Ca = IT 8, Sk = IT 1, He = IT 1. **(B)** Isolate Mut21/06 gave IT: Av = IT 8, Ca = IT 8, Sk = IT 7, He = IT 6c. **(C)** Isolate GB75/30 gave IT: Av = IT 7, Ca = IT 8, Sk = IT 2c, He = IT 6c, and **(D)** Mut15/05 gave IT: Av = IT 7, Ca = IT 8, Sk = IT 6c, He = IT 5–6cn.

Seedlings of the varieties Avocet S and Cartago were susceptible to all four isolates showing IT of seven or above (**Figure 1**). Skater (*Yr*2) and Heines VII (*Yr*2) were resistant to the wild type isolates (IT 1–2). Skater (*Yr*2) was susceptible to both mutant isolates whereas Heines VII (*Yr*2) showed a high intermediate IT (IT = 5–6) with additional necrosis for the isolate Mut15/05.

### Growth of Fungal Colonies in Susceptible and Resistant Wheat Varieties

**FIGURE 2:**
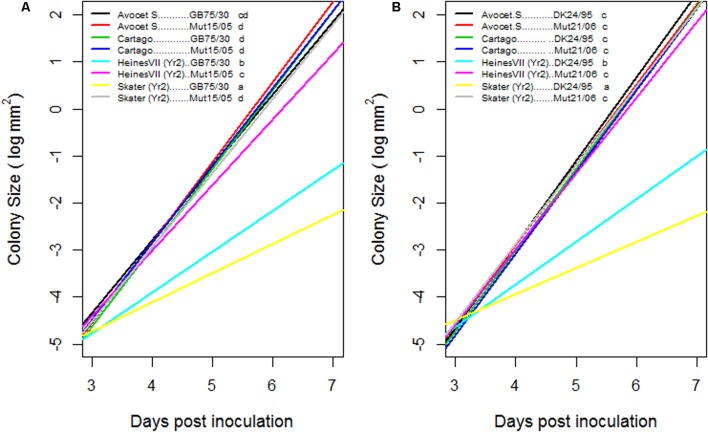
**Estimated growth curves for colony size of two pairs of wild type and mutant isolates GB75/30–Mut15/05** **(A)** and DK24/95–Mut21/06 **(B)**, on seedlings of two wheat varieties Avocet S and Cartago which were susceptible to all four isolates and on Skater and Heines VII with *Yr*2 resistance. *Y*-axis is a logarithmic scale. Growth curves of varieties–isolates combinations followed by the same letter were not statistically significant different (α = 0.05) when adjusted for multiple comparisons.

The increase in colony size as a function of time was significantly different for the compatible interactions compared to the incompatible interactions within the first 7 dai (**Figure 2**). On Skater (*Yr*2) the growth of the virulent mutant isolates was similar to the growth observed on Cartago and Avocet S whereas on Heines VII (*Yr*2) the growth of Mut15/05 was restricted, resulting in a significantly different growth curve compared to the compatible interactions. For the avirulent wild type isolates the increase in colony size was significantly larger on Heines VII (*Yr*2) than on Skater (*Yr*2).

**FIGURE 3:**
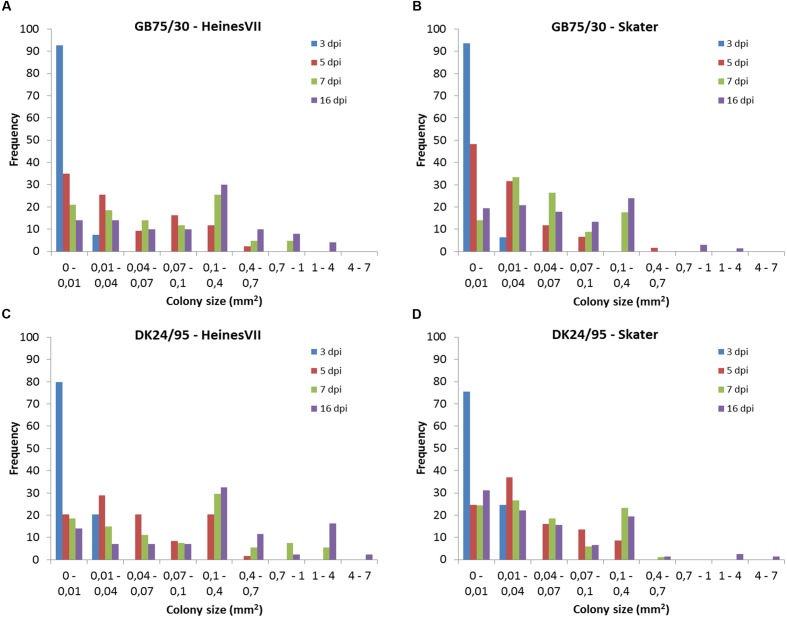
**Frequency distribution for colonies sizes of two avirulent wild type isolates of *Puccinia striiformis*, GB75/30 and DK24/95, at the time points 3, 5, 7, and 16 day post-inoculation (dpi).** Colony size was assessed in the second leaf of seedlings of the two wheat varieties Heines VII and Skater which carries the *Yr*2 resistance gene. **(A)** Colonies of GB75/30 in Heines VII (*Yr*2). **(B)** Colonies of GB75/30 in Skater (*Yr*2). **(C)** Colonies of DK24/95 in Heines VII (*Yr*2). **(D)** Colonies of DK24/95 in Skater (*Yr*2).

The frequency distribution of colony sizes of the avirulent wild type isolates on Skater (*Yr*2) and Heines VII (*Yr*2) showed large overlap between the distributions for different time points (**Figure 3**). In contrast, very little or no overlap was found between distributions for different time points in the compatible interactions (Supplementary Figures S1–S4). The frequency distributions for individual time points within the incompatible interactions were compared by Kolmogorov–Smirnov tests (**Table 2**). All host–pathogen combinations revealed distributions at 3 and 5 dpi that were significantly different from distributions at later time points. The distributions at 16 dpi were in three cases significantly different form the distribution at 7 dpi, which was mainly due to a small percentage of large colonies at 16 dpi. For all four host–pathogen combinations both the variance and the maximum colony size increased with time whereas the smallest observed colonies were similar in size for all time points (**Table 2**). In summary, the results showed that colonies in incompatible interactions were stopped at different time points up to 7 dpi and that a small percentage of colonies continued to grow after 7 dpi.

### Fungal Growth and Host Response during Compatible and Incompatible Interactions

**FIGURE 4:**
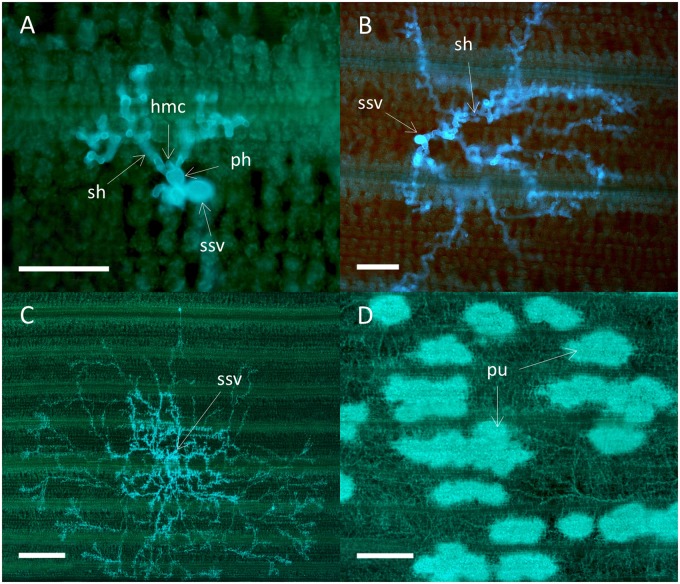
**Colonies of the wild type *P. striiformis* isolate DK24/95 in seedlings leaves of the susceptible wheat variety Avocet S.** Colonies stained with Uvitex 2B were visualized by epifluorescence microscopy. **(A)** Fungal colony with a substomatal vesicle (ssv), two primary infection hyphae (ph) with haustorial mother cells (hmc), and two secondary hyphae (sh) at 3 dpi. Scale bar = 100 μm. **(B)** Fungal colony at 5 dpi. Scale bar = 100 μm. **(C)** A fungal colony with a highly branched mycelium at 7 dpi. Scale bar = 500 μm. **(D)** Hyphal network with several pustules (pu) covering the whole leaf at 16 dpi. Scale bar = 500 μm.

Colony morphology was similar for all four isolates on the susceptible varieties Avocet S and Cartago. At 3 dpi each colony consisted of a substomatal vesicle that had one to two primary infection hyphae with haustorial mother cells. Most colonies also had two to three secondary hyphae (**Figure 4A**). At 5 dpi branched secondary hyphae had developed and started to form a mycelium (**Figure 4B**). A highly branched mycelium was formed at 7 dpi, most often with an oval to radial shape and often with a dense center (**Figure 4C**). Colonies were often overlapping at 7 dpi and at 16 dpi the leaves were densely packed with mycelium networks containing spore bearing pustules (**Figure 4D**). There was no sign of antagonistic effects between overlapping colonies, and colonies were never associated with host cell autofluorescence characteristic for HR. Colony development for virulent mutant isolates in Skater (*Yr*2), were similar to the development on Avocet S and Cartago, i.e., no sign of autofluorescence or necrosis. In Heines VII (*Yr*2), areas with autofluorescence were occasionally detected for the mutant isolate Mut15/05. In addition, small necrotic areas with faint autofluorescence and less densely packed mycelium were observed at 16 dpi.

**FIGURE 5:**
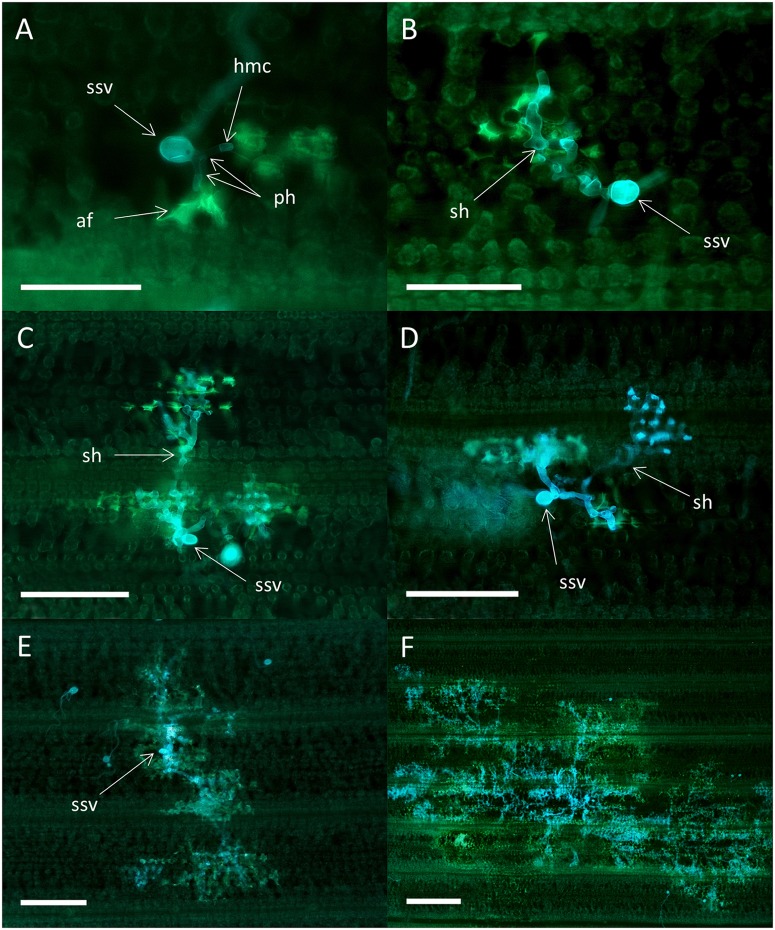
**Colonies of the avirulent wild type *P. striiformis* isolate DK24/95 in seedlings leaves of the resistant variety Skater (*Yr*2).** Colonies stained with Uvitex 2B, and plant host cell response (autofluorescence) were visualized by epifluorescence microscopy. **(A)** Substomatal vesicle (ssv) and two primary infection hyphae (ph) with septate haustorial mother cells (hmc) at 3 dpi. Plant cell autofluorescence (af) in mesophyll cells, indicative of hypersensitive response, are seen close to the hmc. Scale bar = 100 μm. **(B)** Colony with secondary hyphae (sh) closely associated with hypersensitive response at 3 dpi. Scale bar = 100 μm. **(C)** Colony at 5 dpi with three secondary hyphae (sh), all of them associated with high levels of plant cell autofluorescence. Scale bar = 200 μm. **(D)** Colony with four secondary hyphae (sh) at 5 dpi. No autofluorescence are observed for one of the hyphae (arrow). Scale bar = 200 μm. **(E)** Largest type of colony found at 7 dpi. The whole colony appears to be covered with plant cell autofluorescence. Scale bar = 200 μm. **(F)** Largest type of colony found at 16 dpi. Scale bar = 500 μm.

Colonies of the avirulent wild type isolates in Skater (*Yr*2) and Heines VII (*Yr*2) were associated with high levels of autofluorescence in the plant mesophyll cells at all time points, signifying HR (**Figure 5**). In general, the same host reactions and colony morphology were found in both varieties. Small colonies consisting of a substomatal vesicle and one or two primary infection hyphae most often with haustorial mother cells were found at all time points (**Figure 5A**). The largest type of colonies at 3 dpi had formed secondary hyphae (**Figure 5B**). Colonies of both types were also observed at later time points. At 5 dpi, colonies with secondary hyphae of various numbers and degree of branching were the most common, and the colonies were often covered by plant cell autofluorescence (**Figure 5C**). Occasionally, one or two of the secondary hyphae within a colony were not associated with autofluorescence (**Figure 5D**). The largest colonies at 7 dpi (**Figure 5E**) and 16 dpi (**Figure 5F**) had a polarized structure where the center and periphery were associated with high levels of plant cell autofluorescence. All the colony types described above could be observed within the same leaf at 16 dpi.

**FIGURE 6:**
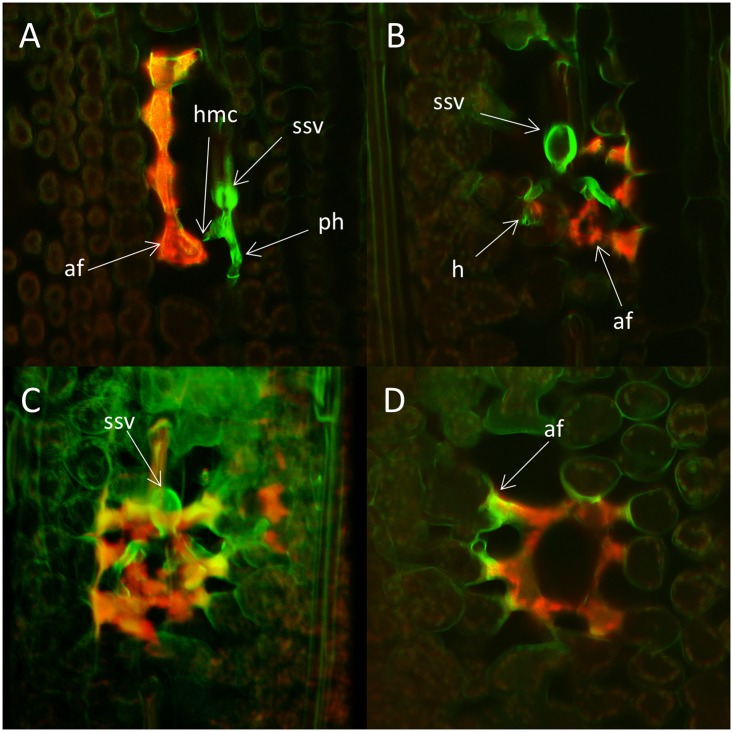
**Colonies of the avirulent wild type *P. striiformis* isolate GB75/30 in seedling leaves of the resistant wheat variety Skater (*Yr*2) 3 and 5 days post-inoculation (dpi).** Colonies stained with Uvitex 2B and plant host cell response were visualized with two-photon and confocal laser scanning microscopy. **(A)** A fungal colony with a substomatal vesicle (ssv) and two primary infection hyphae (ph) with haustorial mother cells (hmc). Strong autofluorescence (af), indicative of hypersensitive response is seen in four plant cells, one of them in close contact with a haustorial mother cell. The shape of the fluorescing plant cells is distorted compared to cells without strong autofluorescence. **(B)** A single z-plane through a colony at 5 dpi. Strong autofluorescence (af) is seen to the right of the colony and a haustorium (h) is visible inside a plant cell. **(C)** 3-D projection of the colony seen in **(B)**. Autofluorescence appear to surrounds the entire fungal colony. **(D)** Plant cells with autofluorescence in a single z-plane underneath the colony in **(C)**. The fluorescing cells are distorted and seem to form some type of network. Strong autofluorescence (af) are seen in the contact zone between distorted and normal cells.

**FIGURE 7:**
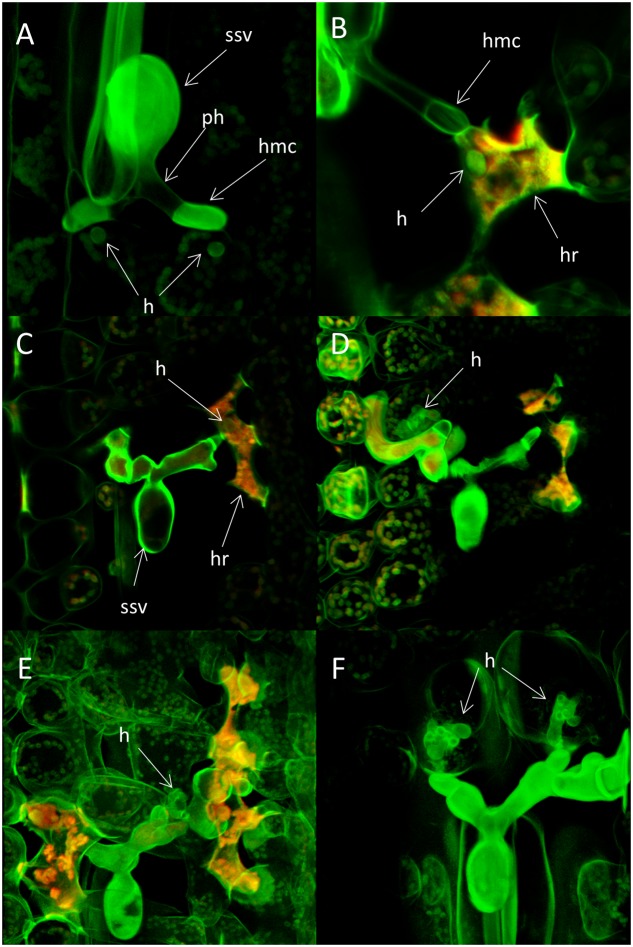
**Haustoria formation and host cell response in seedling leaves of the wheat varieties Heines Peko (*Yr*2) and Kalyansona (*Yr*2) infected with the avirulent wild type *P. striiformis* isolate DK24/95.** Infected seedlings of the susceptible variety Cartago was used as control. Fungal structures stained with Uvitex 2B were visualized with two-photon microscopy. Autofluorescence from plant host cells were visualized by confocal microscopy. **(A)** Fungal colony in Heines Peko 24 h after inoculation (hai). A single immature haustorium can be seen on each of two primary infection hyphae. No sign of host cell autofluorescence. ssv, substomatal vesicle; ph, primary infection hyphae; hmc, haustorial mother cell; h, haustoria. **(B)** A small globose haustorium on a primary infection hyphae 72 hai. Host cell shows hypersensitive response (HR). hmc, haustorial mother cell; h, haustoria; hr, hypersensitive response. **(C)** A fungal colony 120 hai. Host cell with hypersensitive response and a small globose haustorium is present to the right of the ssv. ssv, substomatal vesicle; h, haustorium; hr, hypersensitive response. **(D)** The same colony as in seen **(C)** but in a different confocal plane. A branched haustorium which appear normal in size are seen to the left of the ssv in a host cell with no sign of autofluorescence. **(E)** A colony in the resistant variety Kalyansona 72 hai. A haustorium with a normal appearance is seen in a host cell with no sign of autofluorescence right to the ssv. Autofluorescence are present in other host cells in the close proximity of the colony. **(F)** Two haustoria on the primary infection hyphae 72 hai in seedling leaves of the susceptible variety Cartago.

Colony formation and reaction of host cells during the incompatible interaction were analyzed in more detail by two-photon and confocal laser scanning microscopy (**Figures 6**, **7**). Distorted host cells with autofluorescence, indicative of HR, were observed in close proximity to all fungal colonies after 3 dpi. In most cases, groups of affected cells were observed and at least one of the cells in such a group was in direct contact with a fungal hypha (**Figure 6A**). In these cases, the hypersensitive reaction appeared to have spread from the cells in contact with the fungal structures to the adjacent cells. At 5 dpi some of the cells showing autofluorescence also contained a haustorium (**Figure 6B**). 3-D projections of colonies at 5 dpi showed that often the colony was surrounded by plant cells with autofluorescence, although the resistant response appeared to be highly local only affecting cells in close proximity to the fungal colony (**Figure 6C**). The distorted host cells had a concave shape and seemed to form a kind of network where the plant cell walls were not clearly visible at the point of contact (**Figure 6D**). A very strong autofluorescence signal was often observed in the contact zone between hypersensitive cells and cells that appeared unaffected.

Small globose immature haustoria on the primary infection hyphae were often observed in plant cells prior to any sign of HR (**Figure 7A**). Later, most host cells with a haustorium showed clear sign of HR, and the size and shape of these haustoria were different than haustoria of a compatible interaction (**Figures 7B,C,F**). However, some plant cells contained haustoria that appeared normal in shape and size and no autofluorescence could be recorded for those cells (**Figures 7D,E**).

## Discussion

In this study, we showed that there may be large variation between individual infection sites in an incompatible interaction between *P. striiformis* and a resistant wheat host. We used histological observation to study the temporal and spatial variation of the resistance responses in plants that carries the *Yr*2-resistance. The growth of the avirulent wild type isolates was restricted in the resistant host, probably due to plant cell death (HR) as indicated by autofluorescence and change in the shape of the affected plant cells. The response appeared to depend on initial recognition in cells in direct contact with the fungal structures followed by a spread to adjacent cells and eventually surrounding the fungal colony. Large variation was observed in the time of arrest of individual fungal colonies and in the responses of individual host cells. Overall the interaction appeared dynamic with large temporal and spatial variation. The growth of spontaneous virulent mutant isolates originating from particular wild types, was similar in varieties with *Yr*2 resistance compared to varieties considered fully susceptible, although small deviations were seen.

In the susceptible varieties Cartago and Avocet S, the colony growth of all four pathogen isolates appeared unrestricted. Small differences were seen in IT due to variation in the level of leaf chlorosis but this was not reflected in the growth. The mean colony size increased exponentially within the first 7 dai and colonies had a radial non-polarized shape which is typical for a compatible interaction (Cartwright and Russell, 1981). In the variety Skater (*Yr*2) the growth of the two different virulent mutant isolates were similar to that observed for Cartago and Avocet S, despite small difference in IT. Skater is believed to possess sources of resistance from the variety Carstens V (*Yr*32, +) in addition to the *Yr*2-resistance (Hovmøller, 2007). The resistance in Carstens V appears quite complex and includes several different major and minor genes (Calonnec et al., 2002; Eriksen et al., 2004). Both wild type and mutant isolates gave an IT on Carstens V (results not presented) that was comparable to that of the mutant isolates on Skater. This could indicate that additional resistance shared by these two varieties may have a small effect on the mutant isolates although it does not affect the colony growth. The growth of Mut15/05 was additionally restricted in Heines VII (*Yr*2, +) which was not observed for Mut21/06. The colonies of Mut15/05 were sometimes associated with low level of host cell autofluorescence and necrotic leaf symptoms were seen both at macroscopic and microscopic level. Calonnec et al. (1997b) found that Heines VII in addition to *Yr*2-resistance has one major R-gene, probably *Yr*25 (Boshoff and Pretorius, 1999) and probably also additional resistance with minor effects. They stated that this additional resistance was only expressed against non-European pathotypes, in accordance with the reaction of *Yr*25. The findings here indicate that genetic lineages of yellow rust from the Northwestern-European population may also be affected by additional resistance in Heines VII. The R-gene with major effect, in this case *Yr*2, may therefore mask the effect of minor genes, but as shown here the effect may be disclosed by using an appropriate pathogen isolate.

The two virulence mutant isolates, which were collected on adult plants of Skater in field trials, shared 100% identity with their respective wild type isolate based on AFLP markers (Sørensen et al., 2013). Based on a differential set including three varieties with *Yr*2 resistance (Kalyansona, Heines VII, and Heines Peko) the mutant isolates only differed from the wild types with respect to virulence to *Yr*2. The presence of *Yr*2 in Skater has previously been confirmed based on several well-characterized pathogen isolates (Hovmøller, 2007). A near-isogenic line with *Yr*2 would have been useful, but such a line was not available in the set of Avocet near-isogenic lines developed by Wellings et al. (2009). Instead, the old (year 1950) German variety Heines VII was used. *Yr*2 in Heines VII and Heines Peko has been confirmed in several segregation studies using many pathogen isolates (Lupton and Macer, 1962; Calonnec et al., 1997a,b). Skater is a modern (year 2000) French variety with a different genetic background (http://wheatpedigree.net/). Thus, the differences in growth between the wild type and mutant isolates on Skater and Heines VII were most likely due to a spontaneous mutation in avirulence corresponding to *Yr*2-resistance.

The resistance in Skater and Heines VII against the wild type isolates resulted in smaller colonies that had a polarized shape and were associated with high levels of host cell autofluorescence, typical for a HR. The colony size range and variation increased with time showing that not all colonies were stopped at the same time after inoculation although most colonies appeared to be stopped during the first 5–7 days. Colonies that were not stopped right after the formation of primary infection hyphae or shortly after the formation of secondary hyphae were in many cases observed to have relatively long secondary infection hyphae that were not covered with autofluorescence. A small proportion of the colonies at 16 dpi were very large compared to the rest which shows that few colonies were able to sustain growth much longer than others, although still associated with high levels of autofluorescence.

HR characterized by induced plant cell death that prevents the pathogen from taking up nutrients, is initiated by a burst of reactive oxygen species, which in addition may create a hostile environment for the intruder either by direct toxicity or through establishment of cell wall fortifications (Coll et al., 2011; Mittler et al., 2011). HR is most often combined with release of fluorogenic phenolic compounds (Carver et al., 1994; Heath, 2000) leading to autofluorescence, which has often been reported to correlate with growth restriction of the invading pathogen (e.g., Gousseau and Deverall, 1986; Bender et al., 2000; Münnich et al., 2000). HR is characteristic for resistance based on gene-for-gene interaction (Maor and Shirasu, 2005) and for yellow rust – wheat interaction effects of the *Yr*1, *Yr*5, and *YrSu* R-genes are associated with HR-related gene transcripts (Coram et al., 2008; Yu et al., 2008; Bozkurt et al., 2010). A general delay between the formation of fungal structures and the host response has previously been reported where the extent of the delay depends on the individual R-gene (Wang et al., 2013). Such a delay may explain the large variation in colony size observed in this study. The presence of a significant delay between fungal appearance and host defense response were confirmed by additional observations in the varieties Kalyansona (*Yr*2) and Heines Peko (*Yr*2) showing that colonies were not associated with host cell autofluorescence 24 h after inoculation (hai). A hypothesis could be that the large variation in colony size is because the pathogen is able to detect the presence of HR and subsequently redirect resources to other parts of the mycelium to continue its growth in unaffected leaf areas.

Aspects of host cell responses and colony formation for the incompatible interactions were further investigated by the use of two-photon and confocal microscopy. In particular, two-photon microscopy is well suited for bulky specimens like intact leaves because of deeper penetration and higher resolution compared to single-photon excitation (Feijó and Moreno, 2004). The benefit of advanced microscope techniques is especially evident for studies involving pathogens like *P. striiformis* that mainly grow and interact with the host in cell layers below the leaf epidermis and which cannot be easily genetically transformed due to their biotrophic life style. The resistance responses were highly localized as only host cell close to the pathogen structures became affected, even though the response spread to adjacent cells not in direct contact with the pathogen. This spread may imply some kind of controlled signaling between host cells in the near proximity of the pathogen colony, based on either plant signaling or movement of pathogen effectors (Oliveira-Garcia and Valent, 2015). The affected cells eventually encased the fungal structures and it appeared as if some type of network was induced between them. The hypersensitive host cells appeared distorted with a concave shape, something that has also been observed for epidermal cells of cowpea (*Vigna unguiculata*) resistant to cowpea rust (*Uromyces vignae*) when penetration was attempted (Mellersh and Heath, 2001). Many observations of pathogen–host systems points to the fact that infection leads to changes in the structure and composition of the cell wall of host cells (Underwood, 2012). These changes often include lignification and callose deposition (Ma and Shang, 2004; Wang et al., 2013). Spread of the response to adjacent cells ahead of fungal growth may be necessary to alleviate the effect of the delay between detection and response. The eventual encasement may have two functions, limiting the number cells available for the pathogen to penetrate for uptake of nutrition and to create a direct toxic environment. Although, we saw no sign of toxicity as colonies stopped at an early infection stage appeared normal in leaves sampled at 16 dpi.

Haustorium formation was observed for both compatible and incompatible interactions. Haustoria are defining structures for biotrophic pathogens and they appear to function as interface for uptake of host assimilates and for host–pathogen signaling (Kemen et al., 2005; Voegele and Mendgen, 2011). Onset of HR seems in most cases dependent on the initiation of haustorium formation from which the avirulence product are hypothesized to be secreted (Catanzariti et al., 2007; Yi and Valent, 2013). Our study also indicated an association between the formation of haustoria and HR as formation of haustoria on primary infection hyphae was observed in host cell at 24 hai prior to any sign of HR. Three dai most host cells close to the infection site containing haustoria showed clear sign of HR, and the development of the haustoria was restricted. Interestingly, at later time points some plant cells contained a normal shaped mature haustorium and for these cells no HR could be recorded although other host cells with autofluorescence were found in close proximity. We are not aware that such diverging responses during an R-gene-based incompatible interaction between *P. striiformis* and wheat have been previously reported. It could be hypothesized that it is an effect of bypassing a critical step in the timing of pathogen recognition. For powdery mildew (*Blumeria graminis*) on barley it is well established that individual cells may respond differently to attack even against a compatible isolate (e.g., Lyngkjær et al., 2001; Olesen et al., 2003). Some cells prevent attack by papilla formation or HR whereas others are successfully attacked by the pathogen. The presence of these apparently normal haustoria may be another reason why some colonies can sustain growth longer than others.

Some differences were observed between incompatible reactions on Skater (*Yr*2) and Heines VII (*Yr*2) with higher frequencies of colonies in the smaller size classes on Skater at 16 dpi and more relatively big colonies in Heines VII. Genetic background and environment is known to have a strong effect on expression of *Yr*2 (Calonnec et al., 1997b) but since our experiment was designed to account for environmental effects the observed differences are most likely a result of genetic background. The genetic background of the host may affect the timing of the defense response as several genes are involved in the initiated pathways.

The temporal and spatial dynamics in colony size and host cell response is a potential indicator of the phenotype of the interaction between *P. striiformis* and wheat as different types of response have been observed for different R-genes of either major or minor effect (Moldenhauer et al., 2006; Feng et al., 2008; Bozkurt et al., 2010; Jagger et al., 2011; Zhang et al., 2012). In wheat lines with minor effect R-genes the host response is dependent on the number and type of genes involved. Such minor genes often result in a reduction in fungal colony growth but with less or different effect than observed here for *Yr*2 and with no or highly reduced levels of host plant autofluorescence. Even in varieties that gives a complete response based on pyramiding of several minor genes, the distribution pattern for fungal colony size appears different with, e.g., a continuous increase in the size of the smallest colonies at different time points (Moldenhauer et al., 2006, 2008). A pattern where some colonies appear to continue growing and become relatively big whereas others are arrested shortly after entry was also reported for resistance to stem rust (*Puccinia graminis* f. sp. *tritici*) based on *Sr15* resistance (Gousseau and Deverall, 1986) and in barley against yellow rust (Münnich et al., 2000). So far, these results indicate that the extent of colony size retardation and the colony size frequency distribution are potential parameters for evaluation of the effect and character of host resistance in wheat against *P. striiformis*.

The results in this study emphasize the importance of histological studies for a more complete understanding of host–pathogen interactions. The temporal and spatial variability of the host–pathogen interaction is potentially an important indicator to differentiate between resistance with different modes of action and inheritance. Investigations of the histological landscape of host–pathogen interactions may also have the potential to assist breeding by identification of phenotypes for genetic studies of host resistance. Understanding this variability is also an essential base for interpretation of results generated from physiological, biochemical and molecular studies in general.

## Author Contributions

CS designed the study, carried out the experiment, and analyzed the data. MH designed the trial that generated the mutants and contributed to the design of the study. RL contributed to the statistical analysis. CS, MH, and RL wrote the manuscript. All authors have revised and approved the final manuscript.

## Conflict of Interest Statement

The authors declare that the research was conducted in the absence of any commercial or financial relationships that could be construed as a potential conflict of interest.
