# Supplementary material for: Temporal and Spatial Variability of Fungal Structures and Host Responses in an Incompatible Rust–Wheat Interaction
Source: Front Plant Sci. 2017 Apr 12;8:484. doi: 10.3389/fpls.2017.00484 (PMC5389385; doi:10.3389/fpls.2017.00484)
Supplement: Supplementary file 1 [file Presentation_1.PDF]

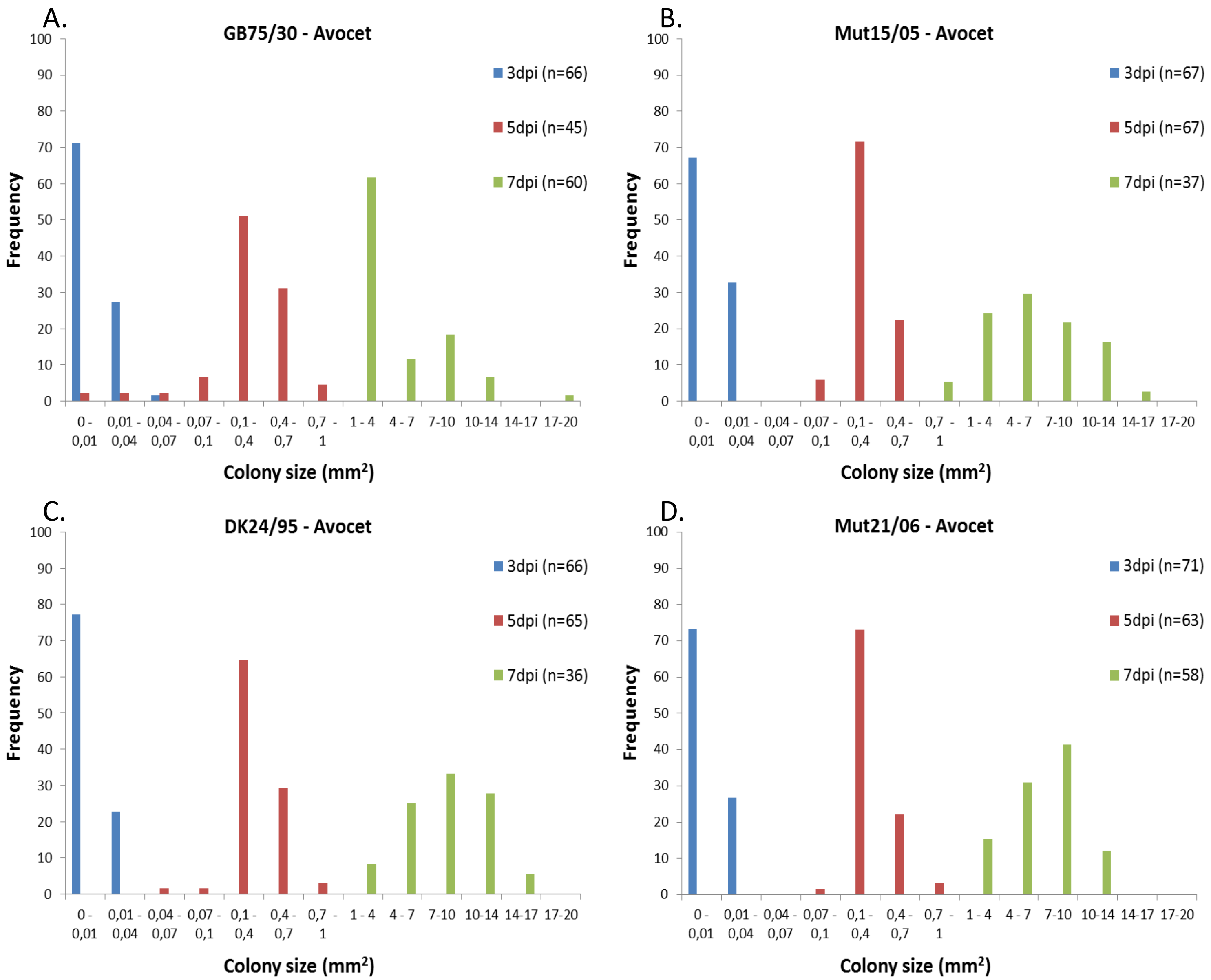

Figure S1. Frequency distribution for colonies sizes of two wild type (GB75/30 and DK 24/95) and mutant isolates (Mut15/05 and Mut21/06) of *Puccinia striiformis* at time points 3, 5, 7 day post inoculation (dpi) in the second leaf of seedlings of the wheat variety Avocet S which is susceptible to all isolates. (**A)** Frequency distribution for GB75/30 (**B)** Frequency distribution of Mut 15/05 (mutant from GB75/30) (**C)** Distribution for DK24/95 (**D)** Distribution for Mut 21/06 (mutant from DK24/95). n = number of colonies assessed per treatment per time point.

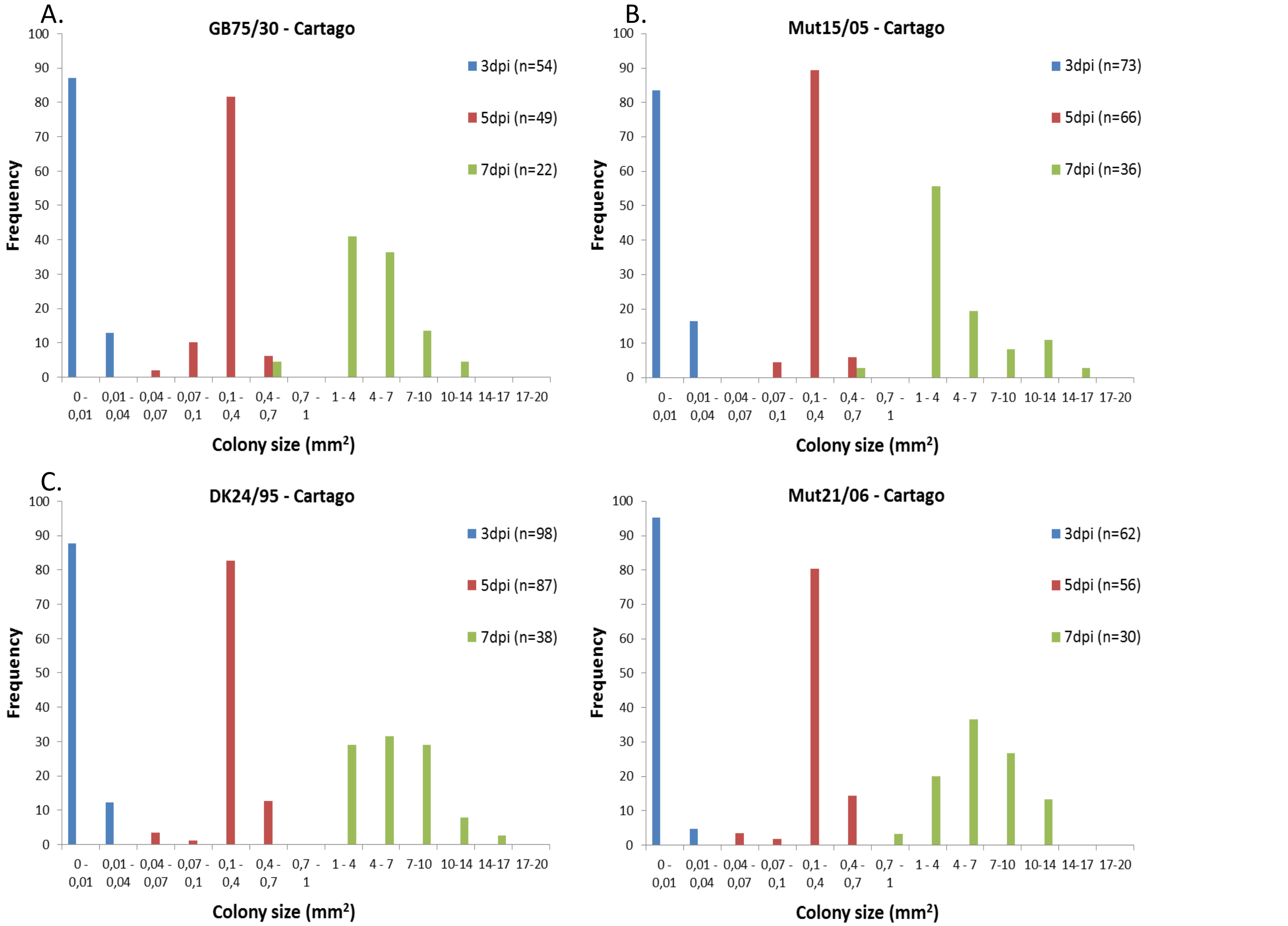

Figure S2. Frequency distribution for colonies sizes of two wild type (GB75/30 and DK 24/95) and mutant isolates (Mut15/05 and Mut21/06) of *Puccinia striiformis* at time points 3, 5, 7 day post inoculation (dpi) in the second leaf of seedlings of the wheat variety Cartago which is susceptible to all isolates. (**A**) Frequency distribution for GB75/30 (**B**) Frequency distribution of Mut 15/05 (mutant from GB75/30) (**C**) Distribution for DK24/95 (**D**) Distribution for Mut 21/06 (mutant from DK24/95). n = number of colonies assessed per treatment per time point.

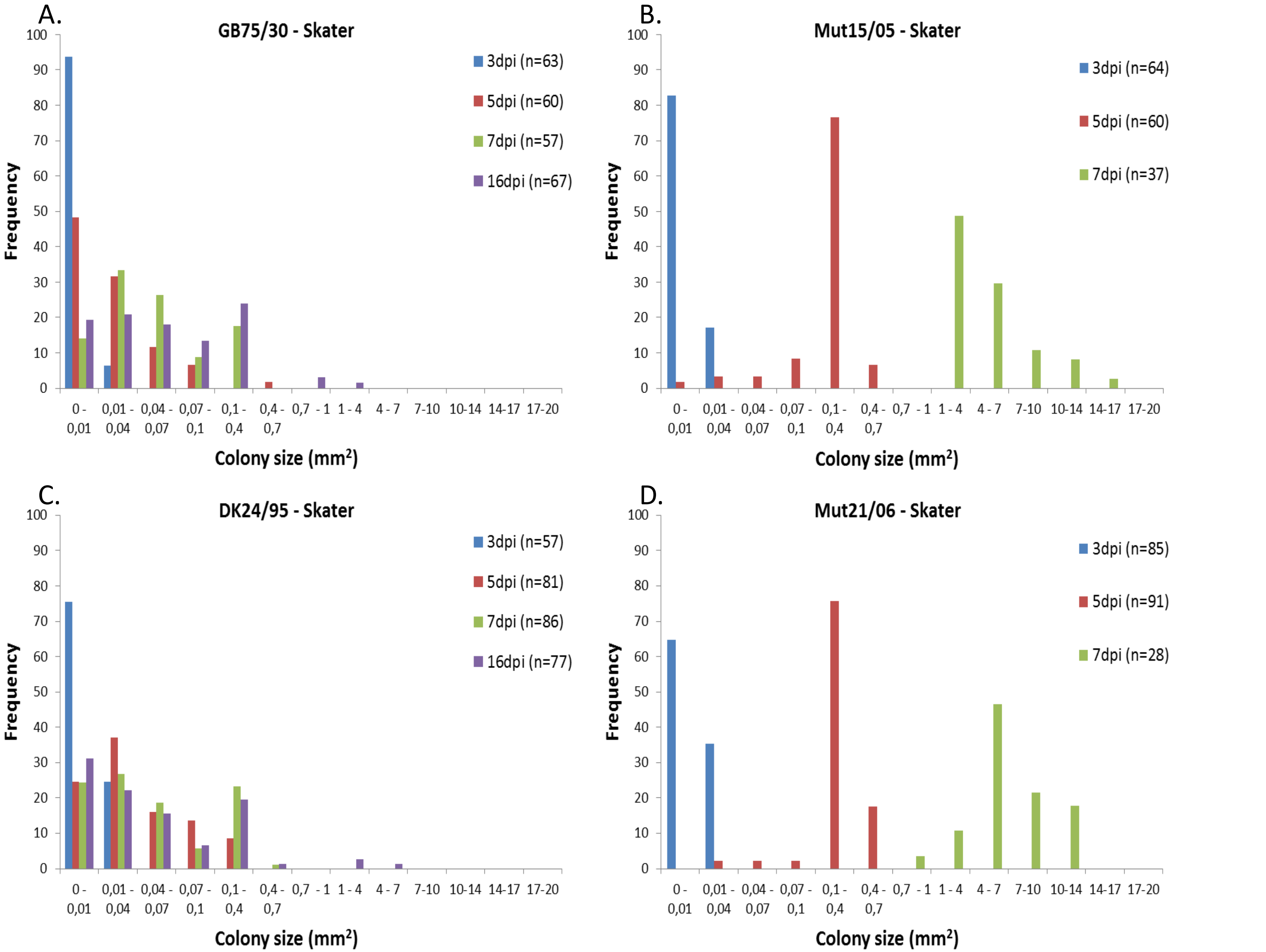

Figure S3. Frequency distribution for colonies sizes of two avirulent wild type (GB75/30 and DK 24/95) and virulent mutant isolates (Mut15/05 and Mut21/06) of *Puccinia striiformis* at time points 3, 5, 7 and 16 day post inoculation (dpi) in the second leaf of seedlings of the wheat variety Skater (*Yr*2) (**A**) Frequency distribution for GB75/30 (**B**) Frequency distribution of Mut 15/05 (mutant from GB75/30) (**C**) Distribution for DK24/95 (**D**) Distribution for Mut 21/06 (mutant from DK24/95). n = number of colonies assessed per treatment per time point.

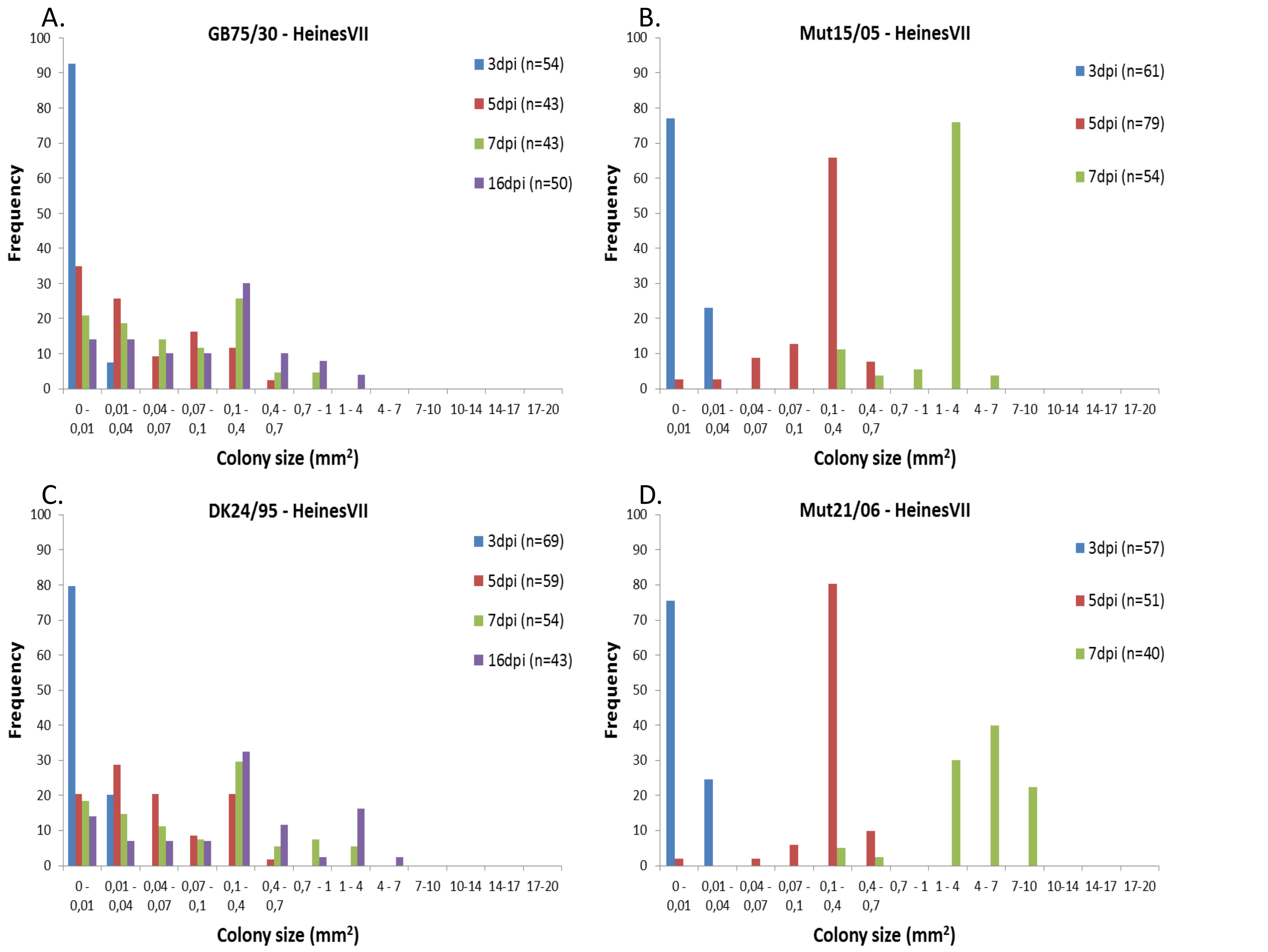

Figure S4. Frequency distribution for colonies sizes of two avirulent wild type (GB75/30 and DK 24/95) and virulent mutant isolates (Mut15/05 and Mut21/06) of *Puccinia striiformis* at time points 3, 5, 7 and 16 day post inoculation (dpi) in the second leaf of seedlings of the wheat variety Heines VII (*Yr*2) (**A)** Frequency distribution for GB75/30 (**B)** Frequency distribution of Mut 15/05 (mutant from GB75/30) (**C)** Distribution for DK24/95 (**D)** Distribution for Mut 21/06 (mutant from DK24/95). n = number of colonies assessed per treatment per time point.
